# Synthetic sex pheromone attracts the leishmaniasis vector *Lutzomyia longipalpis *to experimental chicken sheds treated with insecticide

**DOI:** 10.1186/1756-3305-3-16

**Published:** 2010-03-11

**Authors:** Daniel P Bray, Graziella B Alves, Maria E Dorval, Reginaldo P Brazil, J GC Hamilton

**Affiliations:** 1 Chemical Ecology Group, Institute of Science and Technology in Medicine, Keele University, ST5 5BG, Keele, UK; 2 Departamento de Patologia, UFMS, Campo Grande, Mato Grosso do Sul, Brazil; 3 Instituto Oswaldo Cruz-FIOCRUZ, Av Brasil, 4365 Rio de Janeiro, Brazil

## Abstract

**Background:**

Current strategies for controlling American visceral leishmaniasis (AVL) have been unable to prevent the spread of the disease across Brazil. With no effective vaccine and culling of infected dogs an unpopular and unsuccessful alternative, new tools are urgently needed to manage populations of the sand fly vector, *Lutzomyia longipalpis *Lutz and Neiva (Diptera: Psychodidae). Here, we test two potential strategies for improving *L. longipalpis *control using the synthetic sand fly pheromone (±)-9-methylgermacrene-B: the first in conjunction with spraying of animal houses with insecticide, the second using coloured sticky traps.

**Results:**

Addition of synthetic pheromone resulted in greater numbers of male and female sand flies being caught and killed at experimental chicken sheds sprayed with insecticide, compared to pheromone-less controls. Furthermore, a ten-fold increase in the amount of sex pheromone released from test sheds increased the number of females attracted and subsequently killed. Treating sheds with insecticide alone resulted in a significant decrease in numbers of males attracted to sheds (compared to pre-spraying levels), and a near significant decrease in numbers of females. However, this effect was reversed through addition of synthetic pheromone at the time of insecticide spraying, leading to an increase in number of flies attracted post-treatment.

In field trials of commercially available different coloured sticky traps, yellow traps caught more males than blue traps when placed in chicken sheds. In addition, yellow traps fitted with 10 pheromone lures caught significantly more males than pheromone-less controls. However, while female sand flies showed a preference for both blue and yellow pheromone traps sticky traps over white traps in the laboratory, neither colour caught significant numbers of females in chicken sheds, either with or without pheromone.

**Conclusions:**

We conclude that synthetic pheromone could currently be most effectively deployed for sand fly control through combination with existing insecticide spraying regimes. Development of a standalone pheromone trap remains a possibility, but such devices may require an additional attractive host odour component to be fully effective.

## Background

The sand fly *Lutzomyia longipalpis *(Diptera: Psychodidae) is the principle vector of *Leishmania infantum chagasi *(Kinetoplastida: Trypanosomatidiae), the causative agent of American visceral leishmaniasis (AVL) in Brazil and South America. Transmission occurs through bloodfeeding of female *L. longipalpis *on infected hosts [1], with domestic dogs the primary reservoir in urban and peri-urban areas. Treatment for this fatal human zoonosis is expensive and unpleasant [2], and there is no effective vaccine [3]. As AVL prevalence continues to increase, and the disease spreads further into densely-populated cities [4,5], there remains an urgent need to develop effective methods of controlling transmission, sufficiently affordable to be accessible to Brazilian health authorities and possibly individual communities.

Current strategies to control AVL involve the identification and subsequent culling of infected dogs, and killing sand flies through spraying of aggregations sites with insecticides [6-9]. Culling of domestic pets is unpopular, and while common practice, is difficult to implement effectively [10,11]. Effective control of sand fly populations may have more impact on AVL transmission [8], but while sand flies are broadly susceptible to residual insecticides [7], spraying of aggregation sites close to reported human cases has not proved useful in reducing disease prevalence [12].

The potential efficacy of spraying campaigns could be improved dramatically through exploitation of *L. longipalpis *chemical ecology [13]. Male *L. longipalpis *produce terpene sex pheromones from abdominal glands [14-18], which, in combination with host odour, attract both sexes to aggregations (leks) on or above host animals, where females bloodfeed and are mated [13,19,20]. Conventional treatment of lekking sites (most commonly chicken sheds) with insecticide kills the small number of males that arrive first to establish an aggregation, disrupts pheromone production, and thereby prevents further recruitment (and killing) of significant numbers of both sexes [21]. As a consequence, sand flies may instead seek alternative aggregation sites closer to human habitation, with an associated increase in biting risk and VL transmission [21]. If however, insecticide spraying could be combined with release of pheromone from an artificial source, which could attract both sexes to treated sheds for a sustained period, then potentially large numbers of sand flies could be killed, without risk of aggregations forming elsewhere [13].

Recently, we demonstrated that a synthetic sand fly pheromone, (±)-9-methylgermacrene-B, can be formulated in dispensers and used to attract *L. longipalpis *in the field [22]. The aim of the current study is to determine whether this technology could feasibly be applied to increase the number of sand flies attracted and killed at animal houses sprayed with insecticide. We tested whether more flies were killed at experimental, insecticide-sprayed sheds fitted with pheromone dispensers than no-pheromone controls also sprayed with insecticide, and if the number of flies killed increased with amount of pheromone released. In a small-scale longitudinal experiment, we also investigated whether addition of pheromone can reduce, or even reverse, the decrease in sand fly numbers attracted to chicken sheds that normally occurs as a consequence of insecticide spraying [21], relating number of sand flies killed to pre-treatment levels.

In addition to using pheromone to improve the efficacy of insecticide spraying campaigns, it may also be possible to develop a standalone trap to attract and kill sand flies, without the need for insecticides [23,24]. Similar devices, such as simple sticky traps baited with pheromone, are routinely used in agriculture for monitoring and control of crop pests [25]. In this study, we investigated the feasibility of this approach through a combination of field and laboratory experiments. We carried out several controlled experiments in the laboratory to determine which colour of sticky trap attracted the most females, before testing different coloured traps, with and without pheromone, in the field.

Here, we report results of field trials carried out in Brazil, which explore the feasibility of using synthetic pheromone in combination with both insecticide and sticky traps, as novel approaches for controlling *L. longipalpis*.

## Methods

### Field Study Area

Field studies were conducted in Campo Grande (20° 30' S, 54° 40' W), the state capital of Mato Grosso do Sul, Brazil, between February and June 2009. The affluent centre of this modern city of over 700,000 inhabitants is surrounded by poorer peri-urban areas, with a savannah-like climate characterized by distinct dry and rainy seasons [26]. Sand flies caught in urban and peri-urban areas of Campo Grande are almost exclusively *L. longipalpis*, and belong to the (S)-9-methylgermacrene-B producing chemotype of the species complex [22]. There has been a steady rise in VL prevalence over the last 5 years in both humans and dogs within the city, which has been linked to an increase in *L. longipalpis *abundance [27].

Studies involving insecticide were carried out in a large residential garden of approximately 2000 m^2 ^in the Tijuca district of the city (20° 30'33 S, 54° 39'41 W). Habitat in the garden comprised areas of bare earth, scrub vegetation, and large fruit trees. Chickens (10-20) roosted within trees and an open-fronted roost adjoining a brick wall.

Sticky trap experiments were conducted in existing chicken sheds, constructed from wood, metal sheeting and chicken wire, within residential properties in the Tijuca and Guanandi (20° 30'00 S, 54° 38'42 W) districts of Campo Grande.

### Experimental chicken sheds

Experimental chicken sheds consisted of a 4-sided rectangular plywood enclosure (105 cm [height] by 55 cm [width] by 55 cm [breath]) with a circular 35 cm diameter aluminium lid partially covering the upper opening [22]. Dowling rods were used to contain an individual chicken within the lower part of each shed, with a modified [28] CDC miniature light trap suspended from the lid for sand fly monitoring.

### Insecticide treatment of chicken sheds

Sheds were sprayed with 20 mg a.i.m^-2 ^microencapsulated lambda-cyhalothrin (Karate Zeon, Syngenta, Huddersfield, UK), one of the insecticides recommended to Brazilian health authorities for vector control [29]. Similar formulations of lambda-cyhalothrin have previously been shown to be effective in killing sand flies [30], including *L. longipalpis *[21]. Boxes were resprayed once per week, with chickens, lids and light traps removed to avoid contamination.

### Sticky traps

Commercially available agricultural sticky traps (Russell-IPM Ltd., Deeside, United Kingdom), coated with a dry adhesive, were cut to size for use in laboratory and field experiments. Small traps (60 mm by 60 mm), utilizing both adhesive sides, were used for laboratory cage studies, while single sided, larger traps (250 mm [height] by 250 mm [width]) were used in the field, where traps were placed against chicken shed walls.

### Pheromone dispensers

Pheromone dispensers were those shown previously to attract *L. longipalpis *in the field [22]. Each contained 50 μg of the synthetic sand fly sex pheromone (±)-9-methylgermacrene-B [31]. Control dispensers were identical, but contained no pheromone.

### Pheromone-mediated attraction to insecticide-sprayed experimental chicken sheds

The aim of the first field experiment was to determine whether more sand flies are attracted to (and killed at) insecticide-sprayed experimental chicken sheds fitted with pheromone dispensers, compared to no-pheromone controls. We also tested whether the number of flies attracted and killed could be improved by increasing the amount of pheromone released from ten sheds, simply by using a greater number of dispensers.

Pairs of insecticide-sprayed experimental sheds, designated 'test' and 'control', were placed 3 m apart. At dusk (17:00-19:00), a randomly selected chicken was removed from the garden and placed into each shed. Test sheds had pheromone dispensers (either 1 or 10) attached to the underside of the metal roof using adhesive tape, while control sheds were similarly fitted with a single no-pheromone dispenser.

Modified CDC light traps [28], powered by 6-volt rechargeable batteries, were switched on once the dispensers were in place, and left on overnight to draw flies into attached collecting pots. The next morning (07:00-08:00), pots and dispensers were removed, chickens released, and the numbers and sex of alive and dead flies in each pot determined under ×40 magnification.

Forty pairs of test and control sheds were set out in total, comprising 20 pairs with 1 pheromone dispenser in the test shed, and 20 pairs with 10 dispensers in the test shed. Four pairs were set out per night. The position of test and control shed in each pair was reversed between nights, to control for any potential bias in sand fly numbers arising as a consequence of shed position.

The distribution of sand flies in traps was overdispersed, with a small number of traps catching a relatively high proportion of flies. Data were therefore analysed using generalized linear models (GLMs; [32]) with a log link and negative binomial error structure [33,34], in SPSS 17 (SPSS Inc., Chicago).

To test whether more female flies were caught in test sheds with 1 pheromone dispenser than paired controls without pheromone, number of females in each CDC capture (N = 40) was entered into the model as the dependent variable, with presence of pheromone (yes for captures from test sheds with 1 pheromone dispenser, N = 20; or no for control sheds with 1 control dispenser, N = 20) entered as an independent variable. The significance of pheromone presence as a predictor of number of sand flies caught and killed was then assessed through a χ^2 ^test of change in deviance, following deletion of this term from the model [33]. If the change was significant at P < 0.05, then test and control sheds were found to differ in the number of female sand flies caught. This same process was then repeated to determine if more males were caught in test sheds with 1 pheromone dispenser (N = 20), compared to paired controls without pheromone (N = 20), by entering number of males captured in the model as the dependent variable.

The same modelling procedure was similarly used to determine if test sheds with 10 pheromone dispensers caught more female flies than paired controls with 1 control (no pheromone) dispenser. Number of females captured was entered as the dependent variable (N = 40), with pheromone presence (yes for sheds with 10 pheromone lures, N = 20; no for sheds with 1 control dispenser, N = 20) entered as an independent variable. If deletion of the pheromone presence/absence term from the model resulted in a significant change in deviance (as assessed through χ^2 ^tests as before), then test sheds with 10 pheromone lures differed from paired controls with 1 no-pheromone lure in the number of female sand flies caught. This process was repeated using number of males as the dependent variable, to determine if more males were caught in test sheds with 10 pheromone lures (N = 20) than the paired controls without pheromone (N = 20).

An extension of this approach was used to investigate whether relatively more female flies were caught in test sheds with 10 pheromone dispensers (with respect to the relevant paired control shed without pheromone), compared to those with only 1 pheromone dispenser. Number of female flies captured at each test shed (N = 40) was entered as the dependent variable into a model with a log link and negative binomial error structure as before. Number of female flies captured in the paired control shed was entered as a covariate, in order to control for variation between pairs, with whether the test shed contained 1 (N = 20) or 10 pheromone lures (N = 20) entered simultaneously as a binomial factor. If deletion of the latter term resulted in a significant change in model deviance (as assessed through χ^2 ^tests), then test sheds with 10 pheromone dispensers were found to have differed from those with 1 pheromone dispenser in the number of female sand flies captured, relative to the number caught in the paired (no pheromone) control. This procedure was repeated with number of males captured as the dependent variable, to determine if increasing the number of pheromone dispensers used from 1 to 10 resulted in an increase in number of males captured, relative to paired, no pheromone controls.

### Maintenance of sand fly recruitment post-spraying using pheromone

In the second experiment, we monitored the number of sand flies caught in experimental sheds before and after they were sprayed with insecticide. Our aim was to investigate the extent to which spraying with insecticide disrupted attraction of sand flies to our experimental sheds, and if this effect could be negated, or potentially reversed, through addition of synthetic sex pheromone at the point when sheds were treated with insecticide. This protocol more closely mimics the real life insecticide-treatment of existing chicken sheds, where sand fly aggregations may already be established, albeit on a smaller scale.

Six unsprayed chicken sheds were spaced at least 5 m apart, each fitted with a modified CDC light trap with attached collection pot. At dusk (17:00-19:00), an individual chicken was placed into each shed, and the light trap connected to a 6-volt battery. The following morning (07:00-08:00), chickens were released, and number of flies caught at each shed counted as before. This procedure was then repeated the following night, with the sheds in the same position.

On night three, all sheds were replaced with randomly selected, identical sheds that had been treated with lambda-cyhalothrin. These insecticide sprayed sheds were placed in the same positions previously occupied by unsprayed sheds. This process mimicked shed spraying, while allowing us to keep unsprayed sheds for performing future replicates (direct replacement of unsprayed sheds with those treated with insecticide is referred to as 'spraying' below).

Three of the positioned insecticide sprayed sheds (designated as 'test sheds') were each fitted with 10 pheromone dispensers each. The remaining three insecticide sprayed sheds (designated as 'control sheds') were each fitted with 10 control (no pheromone) dispensers. In an attempt to make pre-spraying abundances between the two groups as equal as possible, sheds were designated as test or control using a matched ranking system, based on number of flies captured over the first two nights. Monitoring of sand flies caught over the two nights following insecticide spraying was conducted as before, with test and control pheromone dispensers replaced each night.

Thirty replicates (15 test sheds with pheromone, and 15 control sheds without pheromone) were carried out in total, in five blocks of six sheds each (3 test and 3 control) over five four-day periods. Sheds were moved between trapping periods, with a three-day break between each trapping session, to reduce the effect of sand flies returning to 'memorized' locations between sets of replicates [13].

A GLM (log link, negative binomial error structure) modelling approach was used to examine whether spraying sheds with insecticide resulted in a significant change in the number of sand flies caught. The first model investigated whether there was an overall difference in the number of female sand flies caught in CDCs in control sheds (i.e., those where pheromone was not deployed, N = 15) before and after insecticide spraying. Total number of females caught at each control shed replicate over two nights was entered as the dependent variable (N = 30), with whether the two-night catch was made before (N = 15) or after (N = 15) insecticide spraying entered as an independent factor. If deletion of the before/after term from the model resulted in a significance change in model deviance (as assessed through a χ^2 ^test), then spraying of sheds with insecticide was found to impact upon number of females caught. The model was rerun with number of males caught over two nights as the dependant variable (30 two-night catches in total) to determine whether insecticide spraying affected number of males captured, comparing captures made before (N = 15) and after (N = 15) spraying as before.

The same modelling process was also used to determine whether insecticide spraying resulted in a change in sand fly captures at 'test' sheds, where pheromone dispensers were deployed alongside insecticide treatment. The two models were run as above, but using two-night captures recorded at test sheds (N = 30, 15 before insecticide and pheromone deployment, 15 after) as the dependent variable, treating males and females separately as before.

The analysis of the dataset was then extended to investigate directly whether there was a difference between the test (pheromone and insecticide) and control (insecticide only) groups, in terms of the change in sand fly numbers caught that occurred with insecticide spraying: i.e. did deployment of pheromone at the same time as insecticide spraying prevent (or even reverse) the decrease in sand flies numbers which is expected to occur as a consequence of insecticide treatment?

To begin, number of female sand flies caught at each shed over two nights after insecticide spraying was entered as the dependent variable (N = 30; 15 test, and 15 control) with number caught in the two nights before spraying at each shed entered as a covariate, in order to control for initial variation in sand fly numbers between sheds. Whether the shed was fitted with pheromone dispensers in conjunction with insecticide spraying was entered as a binomial factor ('test' or 'control'), and the significance of this term in the model assessed though change in deviance following deletion. If the term was significant (χ^2 ^test), then pheromone was found to have an impact on the number of females caught, following spraying of sheds with insecticide.

To investigate the effect of the pheromone on number of males captured, the same model was run again, with number of males caught after insecticide spraying (either alone or in conjunction with deployment of pheromone dispensers) as the dependent variable (N = 30, 15 test and 15 control), and number caught before treatment entered as the covariate. The effect of pheromone was assessed through the significance of the binomial factor ('test' or 'control' i.e. whether or not pheromone was deployed alongside insecticide spraying where the capture was made) as before.

### Laboratory optimisation of sticky traps

Direct choice tests were carried out prior to field trials to investigate which colours of sticky traps are most attractive to sand flies under controlled conditions. Two traps of different colours, each fitted with a single pheromone dispenser in the centre of one of the adhesive sides, were suspended in opposite top corners of netting cages (45 cm × 45 cm × 45 cm). Twenty virgin 5-7 day old female sand flies from our laboratory colony established from Campo Grande [22] were then introduced into the cage through a sleeve which was then tied shut. Humidity within cages was maintained by hanging wet tissue from the exterior cage frame, with the entire apparatus kept within an air-tight white plastic bag for the duration of the experiment. All choice tests were carried out under white light at 26 ± 1 °C, with cages washed with detergent (5% Teepol solution; Sigma-Aldrich Chemicals, Dorset, United Kingdom), rinsed with distilled water and dried between replicates.

Experiments were begun in early evening (5-6 pm), with number of flies caught on each trap recorded the following morning. Colours of traps tested against each other were: white versus yellow, white versus blue, and blue versus yellow. Ten replicates of each pair combination were performed, swapping the positions of each colour within the cage between replicates. Paired t-tests investigated preferences for trap colour by comparing number of flies caught on each trap in each pair.

### Field trials of sticky traps

As blue and yellow traps were found to be the most attractive under controlled conditions, these two colours were selected for trials in the field. Experiments were conducted to determine whether the number of sand flies caught on sticky traps in the field could be increased through addition of a pheromone dispenser, or through use of a particular trap colour. As presence of host odour has been shown to be important in attracting female sand flies to pheromone-baited traps in the field [22], trials were conducted in three occupied chicken sheds within Campo Grande. For each replicate, four different traps were placed in each chicken shed at dusk (17:00-19:00) and left overnight. Two of the traps (one yellow, one blue), had a pheromone dispenser placed in the centre, while the other pair (one yellow, one blue), were each fitted with a control dispenser, containing no pheromone.

Traps were placed 1-1.5 m apart within the shed, and suspended from walls at equal height above ground (1-1.5 m). Number of sand flies of each sex caught on each trap was determined the following morning (07:00-09:00). Thirty six sets of traps were set out in total (12 per shed), with the position of each trap type in each chicken shed rotated between nights, and an equal number of replicates performed with traps in each position. New traps with fresh dispensers were used each night.

As for CDC catches, distributions of male sand flies on sticky traps were overdispersed, and analysed through GLM (log link, negative binomial errors). Number of sand flies caught on each trap was entered as the dependent variable, with trap colour (blue or yellow) and pheromone presence (yes or no) entered simultaneously as factors. Whether colour or pheromone presence made a difference to number of sandflies caught was assessed through χ^2 ^tests of change in deviance following deletion of the relevant term from the model. Numbers of females caught on sticky traps were comparatively low, and were analyzed through Fisher's exact tests.

To determine whether a significant number of female sand flies could be caught on traps that released more pheromone, a second set of experiments were conducted using single pairs of yellow traps, found to be the most attractive colour in the field. One of each pair had 10 pheromone dispensers attached to the sticky surface, while the control had an identical number of control (no pheromone) lures. Pairs were set out in chicken sheds overnight as before, with sex and numbers of sandflies caught determined the following morning. The position of test and control traps was reversed between replicates, with equal numbers of trials carried out with traps in each position. Whether more males were caught on test than control traps was assessed through GLMs as before, with the effect of pheromone presence on numbers caught assessed through change in model deviance following deletion. Numbers of females were again comparatively low, with numbers caught on test and control traps compared through a Fisher's exact test.

### Calculation of means

Count data from field captures were typically overdispersed, and are expressed here as geometric means with 95% confidence intervals [21,35]. To compensate for zeros in data, 0.5 was added to each data point prior to log-transformation, and deducted from the final values for means and confidence intervals post back-transformation. Captures on sticky traps in the laboratory approximated a normal distribution, and are expressed as arithmetic means with 95% confidence intervals for consistency.

## Results

### Pheromone-mediated attraction to insecticide-sprayed sheds

**Figure 1:**
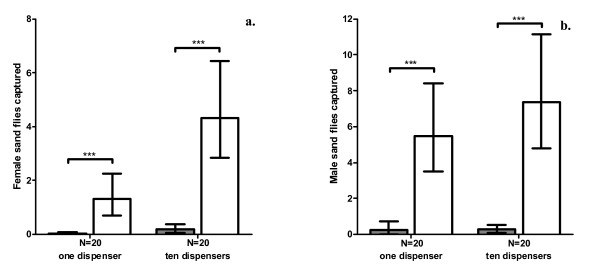
**Sand flies attracted to insecticide-treated chicken sheds using synthetic pheromone**. Number of sand flies (**a**. females; **b**. males; geometric means ± 95% CI) captured and killed at insecticide-treated chicken sheds, fitted with either pheromone dispensers (one or ten, white bars) or one control dispenser (no pheromone, grey bars). * P < 0.05, ** P < 0.01, *** P < 0.001.

Significantly more male and female sand flies were killed at sprayed sheds fitted with a single pheromone dispenser than paired sheds with control (no pheromone) dispensers (females: χ^2 ^= 32.0, df = 1, P < 0.001, Fig 1a; males: χ^2 ^= 36.7, df = 1, P < 0.001, Fig 1b). Similarly, more flies were attracted to test sheds fitted with 10 pheromone dispensers than sheds with a single control (no pheromone) dispenser (females: χ^2 ^= 38.2, df = 1, P < 0.001, Fig 1a; males: χ^2 ^= 50.1, df = 1, P < 0.001, Fig 1b). All flies captured at test and control sheds were dead at counting, with over 90% estimated to have lost one or more legs, indicative of contact with insecticide [7].

Increasing the number of pheromone dispensers from 1 to 10 resulted in a significant increase in the number of females, but not males, trapped and killed at test sheds, relative to paired controls (χ^2 ^= 7.11, df = 1, P < 0.01). In total, the change from 1 to 10 dispensers resulted in a 278% increase in number of female sand flies caught at test sheds (Fig. 1a). No such relative increase was detected for males (χ^2 ^= 1.08, df = 1, P = 0.30; Fig 1b). At the higher rate of pheromone release (10 dispensers), approximately 20 times more flies of both sexes were caught at test stations than controls (Fig 1).

### Maintenance of sand fly recruitment post-spraying using pheromone

**Figure 2:**
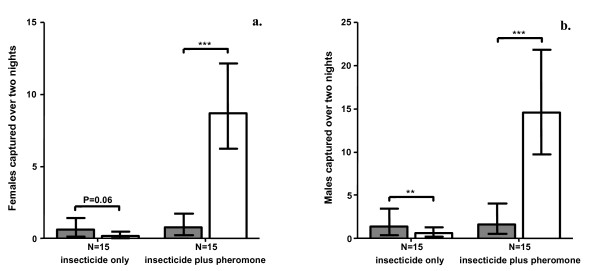
**Effect of pheromone on number of sand flies attracted to chicken sheds following insecticide treatment**. Number of sand flies (**a**. females; **b**. males; geometric means ± 95% CI) captured at chicken sheds over two nights before (grey bars) and after (white bars) treatment with insecticide, applied either with or without synthetic pheromone. * P < 0.05, ** P < 0.01, *** P < 0.001.

Insecticide spraying resulted in a near-significant decrease in number of females captured at control (no pheromone) chicken sheds (compared to pre-spraying levels) over two nights (χ^2 ^= 3.5, df = 1, P = 0.06; Fig. 2a), and a significant decrease in number of males captured (χ^2 ^= 7.7, df = 1, P < 0.01; Fig. 2b). However, at test sheds, where pheromone dispensers were deployed at the same time as insecticide spraying, the number of females and males captured rose significantly above pre-spraying levels (females: χ^2 ^= 18.2, df = 1, P < 0.001, males: χ^2 ^= 13.5, df = 1, P < 0.001,). Separate models confirmed that the change in number of sand flies caught before and after insecticide spraying was significantly altered by the deployment of pheromone (females: χ^2 ^= 38.9, df = 1, P < 0.001, males: χ^2 ^= 37.2, df = 1, P < 0.001). When pheromone was applied in combination with insecticide, 6.3 times as many female sand flies, and 4.4 times as many male sand flies, were caught in the two nights post-spraying compared to the two nights pre-spraying (Fig. 2).

### Laboratory testing of sticky traps

In laboratory choice tests, more female sand flies were attracted to blue traps than white traps (mean females caught: blue = 14.9 (12.84-16.96), white = 1.1 (0.18-2.02), t = 12.0, df = 9, P < 0.001). Similarly, more females were attracted to yellow traps than white traps (yellow = 12.3 (10.61-13.99), white = 4.7 (2.95-6.45), t = 5.6, df = 9, P < 0.001). However, when included in the same test, there was no difference in attractiveness between blue and yellow traps (blue: 8.8 (5.86-11.74) yellow: 9.0 (6.08-11.92), t = 0.08, df = 9, P = 0.9).

### Field testing of sticky traps

**Figure 3:**
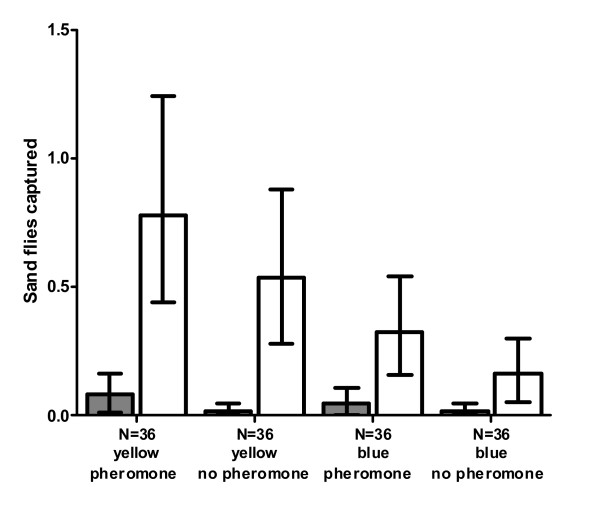
**Sand flies captured on sticky traps of different colours using synthetic pheromone**. Number (geometric mean ± 95% CI) of female (grey bars) and male (white bars) sand flies caught in chicken sheds on blue and yellow sticky traps, with and without pheromone dispensers.

In field trials of traps in chicken sheds, more male sand flies were caught on yellow traps than blue traps (χ^2 ^= 15.0, df = 1, P < 0.01; Fig 3). There was a tendency towards more males being caught on traps with a pheromone dispenser than control traps without pheromone, but this was not found to be significant (χ^2 ^= 3.10, df = 1, P = 0.08; Fig 3).

Considerably fewer female flies were caught on sticky traps than males, with a maximum of 1 female per trap, and only 10 females caught in total. No effect of colour or pheromone presence was found on the distribution of female flies across traps (traps with single female/total traps: blue = 4/72, yellow = 6/72, Fisher's exact test df = 1, P = 0.76; with pheromone = 8/72, without pheromone = 2/72, Fisher's exact test, df = 1, P = 0.10; Fig 3).

In a second round of field experiments, using only yellow traps fitted with 10 dispensers each, pheromone traps caught more males than controls (test: 2.4 (1.24-4.40), control: 0.56 (0.18-1.16); χ^2 ^= 9.70, df = 1, P < 0.01). However, there was still no difference between the number of females caught on traps with and without pheromone (total females caught/total number of traps: test = 4/20 control = 7/20, Fishers exact test, df = 3, P = 0.21).

## Discussion

The presence of synthetic pheromone resulted in greater numbers of sand flies being attracted to, and killed at, insecticide-treated sheds. Furthermore, application of synthetic pheromone with insecticide prevented and reversed the usual decrease in number of sand flies attracted to chicken sheds that normally occurs as a consequence of insecticide treatment [21]. Taken together, these results show that synthetic pheromone can feasibly improve the efficacy of sand fly control programmes, when used alongside existing insecticides. The combination of pheromone and insecticide attracts and kills both sexes, preventing host-seeking females from transmitting VL, and males from establishing alternative aggregation sites elsewhere.

Recruitment of female sand flies to leks is primarily a function of the number of flies present, with recruitment rate increasing with fly abundance [13]. Here, we found that increasing the number of dispensers from 1 (releasing pheromone at approximately the same rate as a group of 50 males [22]) to 10 (500 male equivalents) resulted in an approximately 2.8-fold increase in number of females caught at test sheds. This suggests that females may use amount of pheromone released to assess lek size, and locate larger aggregations. Females initiate and complete copulation more quickly at larger leks, suggesting that they are more receptive to mating when more males are present [36]. However, whether mating in large leks conveys any biological advantage to females is unknown. An alternative, but non mutually-exclusive, explanation is that larger leks may simply be easier to find, as a consequence of the greater amount of pheromone released. Developing a lure that releases greater quantities of pheromone than our current device, and therefore attracts and kills more sand flies, could therefore improve the efficacy of this technology for sand fly control, and its effect on VL transmission. However, there may be an upper limit to release rate, beyond which there is no useful increase in numbers of flies attracted. Further experiments are required to determine the optimum rate of pheromone release from larger, pre-existing chicken sheds, house greater numbers of chickens.

The sex pheromones of *L. longipalpis*, acting in combination with host odour, attracts both sexes to mating aggregations [13]. Here, greater numbers of male sand flies were attracted to test sheds fitted with pheromone dispensers than females. Although males do not blood-feed and transmit VL, manipulating male behaviour is an essential element of effective sand fly control: attracting males to insecticide-treated sheds will prevent them from forming aggregations at alternative sites potentially nearer human habitation, a recognized difficulty associated with spraying animal houses [21]. Immigration rate of males to aggregation sites is dependent upon both number of males present and number of hosts [13]. This may explain why increasing pheromone release at test sites, while keeping number of chickens constant, did not result in an increase in number of males attracted, as occurred with females.

In larger scale studies, the number of sand flies caught at chicken sheds has been found to decrease shortly after insecticide treatment, as the killing of males disrupts pheromone production, and the development of larger aggregations [21]. Here, the number of male sand flies attracted to experimental chicken sheds without pheromone decreased with spraying, accompanied by a near significant decrease in the number of females: the effect on females was most likely limited by the relatively small number caught prior to spraying in our experimental system (a floor effect; [37]. Deploying pheromone simultaneously with insecticide resulted in a 6.3-fold increase in females caught after spraying (compared to before the shed was sprayed), and a 4.4-fold increase in males. While this indicates that synthetic pheromone can override any potential repellent effect associated with insecticide spraying, larger experiments using real chicken sheds, conducted over longer periods of time, are needed to determine the effect that synthetic pheromone, used in conjunction with insecticide, has on pre-existing sand fly aggregations.

Pheromone-baited sticky traps placed inside chicken sheds did not catch more female sand flies than no-pheromone controls, with very few females caught in total. However, more males were caught on test traps compared to controls when the amount of pheromone released was increased from 1 dispenser to 10 dispensers.

Previous field studies have shown that sticky traps can catch both sexes of *L. longipalpis*, but that the pheromone must be used in conjunction with host odour to attract females [22]. While chickens were present in the sheds in which experiments were conducted, it appears that pheromone on its own cannot attract sand flies away from natural leks on nearby host animals. A standalone trap would therefore require a combination of both pheromone and host odour to be released in order to be effective in catching females. Although separate studies have shown that sticky traps baited with hamster odour and pheromone can catch female sand flies in the laboratory [38,39] and some components of host odour attractive to sand flies have been identified [40], an effective pheromone trap combining host odour elements that outcompete natural host/pheromone combinations may require considerable effort to create.

Both sexes of sand flies demonstrated colour preferences, with females preferring blue and yellow traps to white traps in the laboratory, and males preferring yellow traps to blue in the field. Studies of sand fly vision have previously shown that both sexes *of L. longipalpis *are attracted to light in the UV and blue-green-yellow regions of the visual spectrum, although there is some indication that males and females differ in their responses to varying wavelengths [41,42]. Even if colour alone is not an effective attractor of sand flies, these results demonstrate that taking account of colour preferences could aid in fully optimising novel strategies for *L. longipalpis *control.

## Conclusions

Taken together, the results of this study give a clear indication of how pheromone-based technology could most effectively be adopted by sand fly control agencies in the future. Long-lasting pheromone dispensers could be deployed in conjunction with insecticide spraying of chicken sheds, with minimal additional cost in terms of time spent in the field, or additional training. Animal houses are the major foci of nocturnal sand fly activity, with 10 times more sand flies caught in chicken sheds than human houses [43], and are therefore an important target for control [44]. Converting these locations into large-scale, sand fly killing zones could therefore have a dramatic effect on *L. longipalpis *populations, and associated VL transmission rates. If enough synthetic pheromone can be released to attract sand flies away from other potential feeding sites, it may not even be necessary to treat all animal sheds in an area to achieve significant control. While creation of a standalone, pheromone-baited trap is still feasible, work in the immediate future should focus instead on developing pheromone lures that remain effective over a period of several weeks, or months, and measuring the effect of this technology, when used in combination with insecticides, on both *L. longipalpis *populations and incidence of AVL.

## Competing interests

The authors declare that they have no competing interests, other than a patent (No. BRP1050079) for the use of the synthetic pheromone in Brazil held by Keele University, with J.G.C. Hamilton as one of the inventors.

## Authors' contributions

DPB designed and carried out laboratory and field experiments and drafted the original manuscript. GBA and MED carried out fieldwork and reviewed the manuscript. RPB helped with study design, carried out fieldwork and reviewed the manuscript. JGCH conceived the overall study, carried out fieldwork, and drafted and reviewed the manuscript.

## References

[B1] Grimaldi G Tesh RB McMahon-Pratt D A review of the geographic distribution and epidemiology of leishmaniasis in the New World Am J Trop Med Hyg 1989 41 687 725 2701633 10.4269/ajtmh.1989.41.687

[B2] Davies CR Kaye P Croft SL Sundar S Leishmaniasis: new approaches to disease control BMJ 2003 326 377 382 10.1136/bmj.326.7385.377 12586674 PMC1125241

[B3] Noazin S Khamesipour A Moulton LH Tanner M Nasseri K Modabber F Sharifi I Khalil EAG Bernal I Antunes C Smith P Efficacy of killed whole-parasite vaccines in the prevention of leishmaniasis-A meta-analysis Vaccine 2009 27 4747 4753 10.1016/j.vaccine.2009.05.084 19540273

[B4] Maia-Elkhoury ANS Alves WA Sousa-Gomes MLd Sena JMd Luna EA Visceral leishmaniasis in Brazil: trends and challenges Cad Saúde Pública 2008 24 2941 2947 10.1590/S0102-311X2008001200024 19082286

[B5] Desjeux P Leishmaniasis: current situation and new perspectives Comp Immunol Microbiol Infect Dis 2004 27 305 318 10.1016/j.cimid.2004.03.004 15225981

[B6] Lacerda MM The Brazilian leishmaniasis control program Mem Inst Oswaldo Cruz 1994 89 489 495 10.1590/S0074-02761994000300036 7476238

[B7] Alexander B Maroli M Control of phlebotomine sandflies Med Vet Entomol 2003 17 1 18 10.1046/j.1365-2915.2003.00420.x 12680919

[B8] Dye C The logic of visceral leishmaniasis control Am J Trop Med Hyg 1996 55 125 130 8780448 10.4269/ajtmh.1996.55.125

[B9] Lainson R Rangel EF Lutzomyia longipalpis and the eco-epidemiology of American visceral leishmaniasis, with particular reference to Brazil-a review Mem Inst Oswaldo Cruz 2005 100 811 827 16444411 10.1590/s0074-02762005000800001

[B10] Tesh RB Control of zoonotic visceral leishmaniasis: is it time to change strategies? Am J Trop Med Hyg 1995 52 287 292 7694973 10.4269/ajtmh.1995.52.287

[B11] Courtenay O Quinnell RJ Garcez LM Shaw JJ Dye C Infectiousness in a cohort of Brazilian dogs: why culling fails to control visceral leishmaniasis in areas of high transmission J Infect Dis 2002 182 1314 1320 10.1086/344312 12402201

[B12] Amóra SSA Bevilaqua CML Feijó FMC Alves ND Maciel MdV Control of phlebotomine (Diptera: Psychodidae) leishmaniasis vectors Neotrop Entomol 2009 38 303 310 10.1590/S1519-566X2009000300001 19618043

[B13] Kelly DW Dye C Pheromones, kairomones and the aggregation dynamics of the sandfly *Lutzomyia longipalpis* Anim Behav 1997 53 721 731 10.1006/anbe.1996.0309

[B14] Hamilton JGC Sandfly pheromones - their biology and potential for use in control programmes Parasite 2008 15 252 256 18814690 10.1051/parasite/2008153252

[B15] Hamilton JGC Maingon RDC Alexander B Ward RD Brazil RP Analysis of the sex pheromone extract of individual male *Lutzomyia longipalpis *sandflies from six regions in Brazil Med Vet Entomol 2005 19 480 488 10.1111/j.1365-2915.2005.00594.x 16336313

[B16] Hamilton JGC Dougherty MJ Ward RD Sex pheromone activity in a single component of tergal gland extract of *Lutzomyia longipalpis *(Diptera: Psychodidae) from Jacobina, North Eastern Brazil J Chem Ecol 1994 20 141 151 10.1007/BF02065997 24241705

[B17] Lane R Ward RD The morphology and possible function of abdominal patches in males of two forms of *Lutzomyia longipalpis *(Diptera: Phlebotominae) Serie Entomologie Medicale et Parasitologie 1984 22 245 249

[B18] Lane R Phillips A Molyneux DH Procter G Ward RD Chemical-analysis of the abdominal glands of 4 forms of *Lutzomyia longipalpis *- site of a possible sex-pheromone Ann Trop Med Parasitol 1985 79 225 229 4096569 10.1080/00034983.1985.11811912

[B19] Morrison AC Ferro C Pardo R Torres M Wilson ML Tesh RB Nocturnal activity patterns of *Lutzomyia longipalpis *(Diptera, Psychodidae) at an endemic focus of visceral leishmaniasis in Colombia J Med Entomol 1995 32 605 617 7473615 10.1093/jmedent/32.5.605

[B20] Quinnell RJ Dye C An experimental study of the peridomestic distribution of *Lutzomyia longipalpis *(Diptera, Psychodidae) Bull Entomol Res 1994 84 379 382 10.1017/S0007485300032508

[B21] Kelly DW Mustafa Z Dye C Differential application of lambda-cyhalothrin to control the sandfly *Lutzomyia longipalpis* Med Vet Entomol 1997 11 13 24 10.1111/j.1365-2915.1997.tb00285.x 9061673

[B22] Bray DP Bandi KK Brazil RP Oliveira AG Hamilton JGC Synthetic sex pheromone attracts the leishmaniasis vector *Lutzomyia longipalpis *(Diptera: Psychodidae) to traps in the field J Med Entomol 2009 46 428 434 10.1603/033.046.0303 19496409 PMC3197723

[B23] Brazil RP Trumper S Ward RD A heated pheromone trap for the sandfly *Lutzomyia longipalpis *(Diptera: Psychodidiae) Mem Inst Oswaldo Cruz 1989 84 129

[B24] Ward RD Morton IE Brazil RP Trumper S Falcao AL Preliminary laboratory and field trials of a heated pheromone trap for the sandfly *Lutzomyia longipalpis *(Diptera, Psychodidae) Mem Inst Oswaldo Cruz 1990 85 445 452 10.1590/S0074-02761990000400009

[B25] Shani A Chemical communication agents (pheromones) in integrated pest management Drug Dev Res 2000 50 400 405 10.1002/1098-2299(200007/08)50:3/4<400::AID-DDR22>3.0.CO;2-V

[B26] Oliveira AG Galati EAB Oliveira O Oliveira GR Espindola IAC Dorval MEC Brazil RP Abundance of *Lutzomyia longipalpis *(Diptera: Psychodidae: Phlebotominae) and urban transmission of visceral leishmaniasis in Campo Grande, state of Mato Grosso do Sul, Brazil Mem Inst Oswaldo Cruz 2006 101 869 874 17293981 10.1590/s0074-02762006000800008

[B27] Oliveira AG Galati EA Fernandes CE Dorval ME Brazil RP Seasonal variation of *Lutzomyia longipalpis *(Lutz & Neiva, 1912) (Diptera: Psychodidae:Phlebotominae) in endemic area of visceral leishmaniasis, Campo Grande, state of Mato Grosso do Sul, Brazil Acta Trop 2008 105 55 61 10.1016/j.actatropica.2007.09.008 18022137

[B28] Natal D Marucci D Reis IM Galati EAB Modificação da armadilha CDC com testes para coletas de flebotomíneos (Diptera) Rev Bras Entomol 1991 35 697 700

[B29] Secretaria de Vigilância em Saúde Manual de Vigilância e Controle da Leishmaniose Visceral 2003 Brasília: Ministério da Saúde 63 66

[B30] Davies CR Llanos-Cuentas EA Campos P Monge J Leon E Canales J Spraying houses in the Peruvian Andes with lambda-cyhalothrin protects residents against cutaneous leishmaniasis Trans R Soc Trop Med Hyg 2000 94 631 636 10.1016/S0035-9203(00)90214-1 11198646

[B31] Hamilton JGC Bandi KK Mistura racêmica, composto, uso de uma mistura racêmica ou de seu isômero constituinte, métodos de controle ou monitoração de mosquitos-pólvora, de prevenção de infecção e de sintetização de (±)-9-metilgermacreno, e, coleira de animal 2004 BRP10500779

[B32] Crawley MJ GLIM for Ecologists 1993 Oxford: Blackwell Scientific

[B33] Wilson K Grenfell BT Generalized linear modelling for parasitologists Parasitol Today 1997 13 33 38 10.1016/S0169-4758(96)40009-6 15275165

[B34] Wilson K Grenfell BT Generalized linear modelling for parasitologists (vol 13, pg 33, pg 1997) Parasitol Today 1997 13 162 10.1016/S0169-4758(96)40009-6 15275165

[B35] Bland JM Altman DG Transformations, means and confidence intervals BMJ 1996 312 1079 8616417 10.1136/bmj.312.7038.1079 PMC2350916

[B36] Jones TM Quinnell RJ Testing predictions for the evolution of lekking in the sandfly, *Lutzomyia longipalpis* Anim Behav 2002 63 605 612 10.1006/anbe.2001.1946

[B37] Barnard C Gilbert F McGregor P Asking Questions in Biology 2001 2 Harlow: Prentice Hall

[B38] Morton IE Ward RD Response of female sandflies (*Lutzomyia longipalpis*) to pheromone-baited sticky traps in the laboratory Ann Trop Med Parasitol 1990 84 49 51 2331175 10.1080/00034983.1990.11812432

[B39] Oshaghi MA McCall PJ Ward RD Response of adult sandflies, *Lutzomyia longipalpis *(Diptera: Psychodidae), to stick traps baited with host odour and tested in the laboratory Ann Trop Med Parasitol 1994 88 439 444 7979631 10.1080/00034983.1994.11812886

[B40] Dougherty MJ Guerin PM Ward RD Hamilton JGC Behavioural and electrophysiological responses of the phlebotomine sandfly *Lutzomyia longipalpis *(Diptera: Psychodidae) when exposed to canid host odour kairomones Physiol Entomol 1999 24 251 262 10.1046/j.1365-3032.1999.00139.x

[B41] Mellor HE Hamilton JGC Navigation of *Lutzomyia longipalpis *(Diptera: Psychodidae) under dusk or starlight conditions Bull Entomol Res 2003 93 315 322 10.1079/BER2003248 12908917

[B42] Mellor HE Anderson M Hamilton JGC Spectral sensitivity in the eyes of male and female *Lutzomyia longipalpis *sandflies Med Vet Entomol 1996 10 371 374 10.1111/j.1365-2915.1996.tb00759.x 8994140

[B43] Quinnell RJ Dye C Correlates of the peridomestic abundance of *Lutzomyia longipalpis *(Diptera, Psychodidae) in Amazonian Brazil Med Vet Entomol 1994 8 219 224 10.1111/j.1365-2915.1994.tb00502.x 7949312

[B44] Nunes MP Jackson JM Carvalho RW Furtado NJ Courtinho SG Seriological survey for canine cutaneous and visceral leishmaniasis in areas at risk for transmission in Rio de Janeiro where prophylactic measures had been adopted Mem Inst Oswaldo Cruz 1991 86 411 417 10.1590/S0074-02761991000400006 1842432

